# Correlation between Infectivity and Disease Associated Prion Protein in the Nervous System and Selected Edible Tissues of Naturally Affected Scrapie Sheep

**DOI:** 10.1371/journal.pone.0122785

**Published:** 2015-03-25

**Authors:** Francesca Chianini, Gian Mario Cosseddu, Philip Steele, Scott Hamilton, Jeremy Hawthorn, Sílvia Síso, Yvonne Pang, Jeanie Finlayson, Samantha L. Eaton, Hugh W. Reid, Mark P. Dagleish, Michele Angelo Di Bari, Claudia D’Agostino, Umberto Agrimi, Linda Terry, Romolo Nonno

**Affiliations:** 1 Moredun Research Institute, Pentlands Science Park, Bush Loan, Penicuik, EH26 0PZ, United Kingdom; 2 Department of Food Safety and Veterinary Public Health, Istituto Superiore di Sanità, Viale Regina Elena 299, 00161, Rome, Italy; 3 Animal and Plant Health Agency (APHA -Weybridge), New Haw, Addlestone, Surrey, KT15 3NB, United Kingdom; 4 Animal and Plant Health Agency (APHA-Lasswade), Pentlands Science Park, Bush Loan, Penicuik, EH26 0PZ, United Kingdom; Creighton University, UNITED STATES

## Abstract

The transmissible spongiform encephalopathies (TSEs) or prion diseases are a group of fatal neurodegenerative disorders characterised by the accumulation of a pathological form of a host protein known as prion protein (PrP). The validation of abnormal PrP detection techniques is fundamental to allow the use of high-throughput laboratory based tests, avoiding the limitations of bioassays. We used scrapie, a prototype TSE, to examine the relationship between infectivity and laboratory based diagnostic tools. The data may help to optimise strategies to prevent exposure of humans to small ruminant TSE material via the food chain. Abnormal PrP distribution/accumulation was assessed by immunohistochemistry (IHC), Western blot (WB) and ELISA in samples from four animals. In addition, infectivity was detected using a sensitive bank vole bioassay with selected samples from two of the four sheep and protein misfolding cyclic amplification using bank vole brain as substrate (vPMCA) was also carried out in selected samples from one animal. Lymph nodes, oculomotor muscles, sciatic nerve and kidney were positive by IHC, WB and ELISA, although at levels 100–1000 fold lower than the brain, and contained detectable infectivity by bioassay. Tissues not infectious by bioassay were also negative by all laboratory tests including PMCA. Although discrepancies were observed in tissues with very low levels of abnormal PrP, there was an overall good correlation between IHC, WB, ELISA and bioassay results. Most importantly, there was a good correlation between the detection of abnormal PrP in tissues using laboratory tests and the levels of infectivity even when the titre was low. These findings provide useful information for risk modellers and represent a first step toward the validation of laboratory tests used to quantify prion infectivity, which would greatly aid TSE risk assessment policies.

## Introduction

Scrapie is a member of the transmissible spongiform encephalopathy (TSE) family of diseases, or prion diseases which naturally affects sheep, goats and mouflons [1]. Scrapie is transmissible experimentally to several species, including ruminants and rodents, but there is no evidence of transmission to transgenic mice expressing human prion protein (PrP) [2], and there is no epidemiological evidence to suggest that scrapie could pose a risk to public health. However, there is the possibility that the bovine spongiform encephalopathy (BSE) agent, of proven zoonotic potential, may have entered goat herds in the UK [3] and France [4] and possibly also sheep flocks. It is therefore important to be able to detect any ovine TSE infectivity in tissues that might enter the human food chain and in those that might lead to excretion of the infectious agents onto sheep grazed pastures.

TSEs including scrapie are characterised by the accumulation of a pathological form of a host encoded sialoglycoprotein known as prion protein (PrP). Since the link between the normal cellular prion protein (PrP^c^) and TSEs was established [5], several operational terms have been used for the pathological form (abnormal PrP), including PrP scrapie (PrP^Sc^), protease-resistant PrP (PrP^res^), mainly used for Western blot (WB) results and disease-associated prion protein (PrP^d^) used for immunohistochemistry (IHC). There are important differences between animal species in the distribution of the pathological prion protein isoform in TSEs; in field cases of BSE in cattle it is restricted to the central and enteric nervous systems (CNS and ENS, respectively), whereas in both natural scrapie and experimental sheep BSE abnormal PrP is also found in the lymphoreticular system (LRS) and the peripheral nervous system (PNS) [6].

In order to minimise the risk of TSE agents entering the human food chain specified risk material (SRM) is removed from the carcasses of ruminants at slaughter and destroyed [7]. Whilst this approach minimizes the risk based on current knowledge, precise understanding of the distribution of infectivity in several tissues and organs of TSE infected small ruminants is incomplete.

Infectivity loads in tissues from scrapie affected sheep is still partially based on empirical data generated from experimental titrations carried out and published in the early 1980s [8], prior to the discovery of the role played by the PrP gene (*Prnp*) in TSEs [7]. More recent studies on infectivity and detection of abnormal PrP have been carried out in sheep of different *Prnp* genotypes [9–11], yet crucial information on several tissues from highly susceptible sheep of the ARQ/ARQ genotype (where A, R and Q stand for alanine, arginine and glutamine at codons 136, 154 and 171, respectively) is still lacking [7]. Quantification of infectivity in tissues from natural scrapie cases in sheep of this genotype has been hampered because some of those isolates do not readily transmit to mice. Therefore, data regarding the distribution of infectivity in tissues of ARQ/ARQ sheep is mostly extrapolated from the results of laboratory tests for abnormal PrP. However, whereas the latter is believed to be the most useful marker of TSE disease identified to date, it has also been shown that its presence does not always directly correlate with infectious titres [12] and bioassay is still required for verification.

Recently, the bank vole (*Myodes glareolus*) has been shown to be highly sensitive to some sources of natural scrapie that do not readily transmit to mice [13]. In the present study, the vole bioassay was used to determine the distribution of scrapie infectivity in ARQ/ARQ Suffolk sheep from a naturally affected intensely studied flock [14]. The distribution of infectivity was then compared with the results of laboratory tests for abnormal PrP including IHC, WB and enzyme-linked immunosorbent assay (ELISA), as the most commonly used in TSE diagnostics. Furthermore, protein misfolding cyclic amplification (PMCA), a method for abnormal PrP detection based on the *in vitro* conversion of PrP^c^, was applied to selected samples; this test is becoming increasingly used because of its sensitivity [15].

In summary, the aim of the study was to compare the distribution of infectivity and disease associated prion protein in tissues of scrapie-affected sheep, with specific emphasis on those with potential to enter the food chain; in so doing, we have tried to ascertain the predictive value of *in vitro* laboratory test results in determining infectivity.

## Materials and Methods

### Sheep samples

Four 22–28 month old Suffolk sheep of the ARQ/ARQ scrapie susceptible *Prnp* genotype, clinically affected with natural scrapie (animal numbers: B1216, B1217, B1221, B1223) from a well studied and documented flock [14,16] and 4 age-matched, ARQ/ARQ, New Zealand derived, scrapie free sheep (animal numbers: N523, N449, N456, N506) were culled by electrical stunning followed by exsanguinations. At post-mortem a wide range of tissues was collected, including: brain, trigeminal, stellate and thoracic sympathetic chain ganglia, sciatic and vagal nerve trunks, 3^rd^ eyelid, recto-anal mucosal associated lymphoid tissue (RAMALT), distal jejunal lymph node (LN), prescapular LN, semitendinosus, oculomotor (ventral oblique and rectus dorsalis) and tibialis cranialis muscles, liver, adrenal gland, tongue, ileum (including Peyer’s patches and ENS), heart, lung, kidney and pancreas.

All animal procedures complied with the Animals (Scientific Procedures) Act 1986 and were approved by the Moredun Research Institute ethics committee.

Each tissue sample was bisected and one piece immersed in 10% buffered formalin for histopathological and IHC examinations and a second piece processed under sterile condition to a homogenous tissue macerate to maximise homogeneity of abnormal PrP/infectivity in the sample for each subsequent assay. Each tissue macerate was then divided into four separate sterile tubes for future analyses (WB, ELISA, PMCA and bioassay) and stored at −80°C until required.

### Histology and Immunohistochemistry

All tissues from all sheep were processed by routine procedures and embedded in paraffin wax, sectioned (5μm), mounted on glass microscope slides and stained with haematoxylin and eosin or subjected to IHC labelling for abnormal PrP using primary antibody R145 as described previously [17].

Brain tissue was examined for the presence of vacuolation and the magnitude and distribution of abnormal PrP was scored in all tissues. The abundance of abnormal PrP in each visceral tissue was subjectively scored from 0–3 where 0 corresponded to no labelling detected and 1, 2 and 3 corresponded to mild, moderate and severe accumulations of abnormal PrP, respectively. As muscle samples showed abnormal PrP labelling in muscle spindles only and not in myocytes, a semi-quantitative scoring system was applied where scores of 0, 1, 2, 3 corresponded to 0, 1, 1–5, >5 positively labelled muscle spindles, respectively.

### Western blot

Tissue samples (Table 1) from four scrapie-infected and one uninfected control sheep were tested for abnormal PrP by Western blotting.

**Table 1:** Proportion of sheep testing PrP^Sc^ positive in each tissue by three Western blot methods.

	BioRad	Centrifuge	NaPTA
	no. positive/ no. tested	no. positive/ no. tested	no. positive/ no. tested
**Brain**	4/4	4/4	4/4
**Dist. Jej LN**	4/4 (49)	4/4 (23)	4/4 (30)
**Prescap. LN**	4/4 (44)	4/4 (19)	4/4 (18)
**Oculo. ms**	3/4 (5)	3/4 (7)	2/4 (3)
**Sciatic**	1/4 (15)	3/4 (12)	1/4 (7)
**Kidney**	2/4 (12)	1/4 (9)	1/4 (5)
**Tongue**	neg	neg	neg
**Heart**	neg	neg	neg
**Liver**	neg	neg	neg
**Pancreas**	neg	neg	neg
**Semitend. ms**	neg	neg	neg
**Cran. tib. ms**	neg	neg	neg

Number in parentheses is the mean relative intensity (%) compared with 1/8 diluted brain sample.

Approximately 200mg of each tissue examined was weighed into a ribolyser tube and 4 volumes of 0.1M Tris pH8.0/0.1M NaCl buffer added. Tissues were disrupted at 6.5m/s x 40 sec for two cycles using the Fast Prep machine (FP120, Bio101 Thermo Electron Corporation). Tissue homogenates were used in the comparison of three sample preparation methods as follows:

#### Centrifugal concentration

300μl of each tissue homogenate were shaken at 37°C with 2% sarkosyl solution prior to treatment with proteinase K solution (50μg/ml) for 1h at 37°C with shaking (1200rpm, Eppendorf Thermomixer). Digestion was terminated by adding Pefabloc (SC Roche) to a final concentration of 1mM. Samples were then centrifuged at 20,000×g for 1h at 10°C and pellets resuspended in 50μl 2x Sample Buffer (Invitrogen) and heated at 100°C/5min prior to SDS-PAGE. A dilution series of 50%, 25% and 12.5% was made from the resuspended brain sample of each scrapie-infected sheep and included on each gel to act as a positive control and to provide a range of intensities (standard curve) to which the other tissue signals could be compared.

#### NaPTA precipitation

Tissue homogenate (0.5ml) was made up to 1ml with 4% sarkosyl in Dulbecco’s PBS and shaken at 37°C for 10min. Benzonase (50U/ml final concentration) and MgCl_2_ (1mM final concentration) were added prior to shaking at 50°C for 30min. Proteinase K (PK) was added to a final concentration of 50μg/ml and incubated at 50°C for 60min prior to addition of Pefabloc (1mM final concentration) to terminate digestion. Pre-warmed (37°C) NaPTA solution was added to a final concentration of 0.4% and shaken at 37°C for a further 30 min. Samples were then centrifuged at 20,000g for 30min at 10°C and the resulting pellets washed in 0.1% sarkosyl/PBS and 250mM Edetic Acid and re-pelleted at 20,000g /15min at 10°C. Final pellets were resuspended in 50μl 2xSB (Invitrogen) and heated at 100°C/5min prior to SDS-PAGE. A dilution series of 50%, 25% and 12.5% was made from the resuspended brain sample as before.

#### SDS-PAGE and Western blotting

Only one animal’s set of tissues were analysed on each gel to allow direct comparison of intensity between tissues. 10μl of sample was loaded onto 12% Bis-Tris NuPAGE gels (Invitrogen) run at 150V for 1h. Proteins were electrotransferred onto PVDF membrane at 30V for 1h, then subsequently placed in 5% non-fat milk/Tris Buffered Saline Tween 20 (TBST) to block non-specific binding prior to being probed with antibody P4 (R-biopharm, 1:2000 dilution in TBST). Secondary antibody (ECL Mouse IgG, HRP-linked F(ab')₂ fragment from sheep, Amersham) was applied at 1:20,000 dilution in TBST for 1h. Immunoreactivity was detected using Pierce WestFempto chemiluminescent reagents and a Kodak IS440 image station.

#### BioRad TeSeE Western blot

All tissue samples were tested using the BioRad TeSeE WB Sheep and Goat kit according to the manufacturer’s instructions. The only variable introduced was the resuspension of some lymph node samples with large final pellets into two volumes of sample buffer prior to loading. A dilution series of 50%, 25% and 12.5% was made from the resuspended brain sample as before.

#### Image analysis

Western blot images were captured using Kodak 1D 3.6 software. Band analysis was carried out and the mean intensity of abnormal PrP bands was measured. To allow for gel to gel variation, only samples from the same gel were compared and quantitatively analysed. The mean intensities of the edible tissue samples were compared with the mean intensity value of the 12.5% dilution brain sample on the same gel as this sample gave consistent intensity values without signal saturation, which occurred at higher concentrations.

### ELISA

The Bio-Rad TeSeE BSE diagnostic test was used as directed by the manufacturers with modifications to take into account the different consistencies of the tissues and levels of PrP^c^ in the uninfected tissues. Tissues were ribolysed, using a single large (6mm diameter) ceramic bead, to give 20% (w/v) homogenate. Samples were treated with DNAse following ribolysation. Following precipitation and centrifugation at 15,000g for 7min, the resultant pellets were solubilised by incubating in Reagent C at 100°C for 5min. Thereafter the method was followed exactly as per the manufacturer’s instructions. Absorbance was read at 450nm and 620nm using a Victor multi-well plate reader (Perkin-Elmer).

To determine the lowest concentration of PK for each tissue where PrP^c^ was undetectable by the assay, samples from three unexposed control sheep were treated with decreasing concentrations of PK. To determine the relative concentrations of PrP^c^ in each tissue we also assayed for PrP content in the absence of PK. The relative amounts of undigested PrP observed correlated with those published by Moudjou et al. (2001) and the range of PK concentrations required to digest all PrP^c^ was from 0.086 units/ml (prescapular LN) to 1.72 units/ml (tongue, kidney and semitendinosus muscle). When spiked with scrapie positive brainstem and digested using the pre-determined concentration of PK, each matrix (n = 2) gave absorbance readings above the designated assay cut-off point and the matrix did not significantly interfere with the detection of abnormal PrP.

For the determination of abnormal PrP in the absence of PK, the “IDEXX Herd-Chek BSE/Scrapie Antigen EIA Test” was carried out according to the manufacturer’s instructions with some modifications. In brief, the samples were homogenised as described but with the incorporation of a single large (6mm diameter) ceramic bead and by performing three agitation cycles at 6.5ms^-1^ each for 45 seconds; the homogenates were cooled between each cycle. This facilitated better homogenisation of tissues. The samples were mixed with the working plate diluents, loaded on to the antigen-capture plate and shaken for 150min at room temperature. Abnormal PrP was detected using the kit-conjugated anti-PrP antibodies, visualised with TMB and absorbance read at 450nm and 620nm. For end-point dilution the samples were serially diluted in normal brain homogenate prior to PK digestion.

Threshold values for each individual tissue were also calculated. Eight tissues (excluding brain) from ARQ/ARQ negative control sheep (n = 15), from the AHVLA TSE-free flock, were assayed using both modified protocols and the mean absorbance calculated. This mean absorbance value +3 standard deviations was used as the cut-off values above which the sample was considered to be positive.

### Bioassay in bank voles

Brain, sciatic nerve, prescapular lymph node, kidney, oculomotor and semitendinosus muscle, heart and tongue from B1223 and B1213 were used for this part of the study having showed the larger number of positive samples by IHC. All samples were homogenised to 10% w/v (10^−1^ dilution) in phosphate buffered saline (PBS). For the brain inocula, serial tenfold dilutions in PBS (10^−1^–10^−6^) were prepared and used for end point titration into voles.

Groups of 15–20 eight-weeks-old bank voles (*Myodes glareolus*) were injected by the intracerebral route with 20μl of 10% homogenates of edible tissues or serial dilutions of brain inocula into the left cerebral hemisphere under ketamine (0.1 μg/g) anaesthesia. Bank voles have a polymorphism at codon 109 of the PrP gene, coding for either methionine or isoleucine, and this amino acid variation influences the susceptibility of voles to different prion strains [18,19]. All voles used in this study were homozygous for methionine at codon 109 as they are very sensitive to the source of scrapie used here [13]. Beginning 1 month after inoculation, animals were examined twice per week until the appearance of neurological signs from which point they were examined daily. Diseased animals were culled (using a rising concentration of carbon dioxide) at the terminal stage of disease but before neurological impairment was such that it compromised welfare and, especially, adequate drinking and feeding. Survival time (days post-infection, dpi) was calculated as the interval between inoculation and culling. ID_50_ per gram were determined by end point titration according to the Spearman and Kärber method [20].

All the bioassays in bank voles were concluded between 735 and 868 dpi, by culling all the voles that were still alive at that time. Survivors were animals alive until the end of the experiment with no signs of infection. Animals culled for intercurrent disease at <200 dpi were all negative by WB and were excluded from analysis. The attack rate was calculated as the ratio of voles confirmed positive over the number of voles inoculated, excluding animals culled for intercurrent disease before 200 dpi.

The brains of voles culled at the terminal stage of disease, for intercurrent disease or at the end of the experiments, were collected. Each brain was cut parasagitally into two parts, the smaller one stored at −80°C for biochemical studies, the other fixed in 10% buffered formalin for histological assessment of TSE-specific spongiform degeneration analysis as described previously [21]. PK-resistant PrP was examined by Western blotting in SDS-PAGE gels, as previously described [21].

All the procedures used in this study have been approved by the Service for Biotechnology and Animal Welfare of the Istituto Superiore di Sanità and authorized by the Italian Ministry of Health, according to Legislative Decree 116/92, which implemented the European Directive 86/609/EEC on laboratory animal protection in Italy. Bank voles (Istituto Superiore di Sanità breeding colony) were housed in standard cages and treated according to Legislative Decree 116/92 guidelines, and animal welfare was routinely checked by veterinarians from the Service for Biotechnology and Animal welfare. All animals were individually identified by a passive integrated transponder.

### Western blot analysis of the inocula

Abnormal PrP contained in the inocula used to infect voles was detected by WB using a method published previously, which showed high sensitivity for abnormal PrP detection in peripheral tissues [22]. For WB analysis of the inocula, 1 ml of tissue homogenates (10% w/v in PBS pH 7.4) were added to 110l of PBS containing 20% Sarcosyl (Sigma) to obtain a final Sarcosyl concentration of 2% and incubated for 20min at 37°C with gentle shaking before PK (50g/ml) digestion for 60min at 37°C with gentle shaking. Protease treatment was stopped with phenylmethylsulfonyl fluoride (PMSF, Sigma) (33μl of a stock solution, to give a final concentration of 3mM) and the treated homogenates were centrifuged at 20,000g for 60min at 20°C. Pellets were dissolved in 40μl of NU-PAGE sample buffer (Invitrogen), heated at 95°C for 10min and centrifuged in a microcentrifuge for 5min at 8,000g. Twenty microlitres of the resultant supernatants (50mg tissue equivalent per lane) were loaded onto 12% bis-Tris polyacrylamide gels (Invitrogen). Electrophoresis was carried out at 200V for 40min and WB performed on PVDF membranes (Millipore). The blots were blocked for non-specific binding in PBS containing 0.1% Tween_20_ and 3% non-fat milk powder for 1h. Abnormal PrP was detected with the monoclonal antibody P4 (0.4μg/ml, R-Biopharm) for 60min at room temperature. Peroxidase-conjugated goat anti-mouse IgG (1:80,000 for 1h, Pierce) was used as secondary antibody. The membranes were developed with the SuperSignal West Femto enhanced chemiluminescence method (Pierce). Chemiluminescence was detected with the VersaDoc imaging system (Bio-Rad).

The experiment was repeated 3 times and in all experimental sessions the respective brain dilutions were analysed in comparison with the edible tissues.

### PMCA

Brain, sciatic nerve, tongue, prescapular lymph node, heart, kidney, semitendinous and oculomotor muscels from sheep B1216 were examined in this part of the study. For the preparation of substrates, voles carrying M109M PrP genotype [19] were culled using carbon dioxide and immediately perfused with phosphate-buffered saline (PBS) containing 5mM ethylendiaminetetraacetic acid (EDTA). Immediately after perfusion, the brains were removed from the skull and frozen at −80°C. Brain homogenates (10% w/v) were prepared in conversion buffer (PBS, pH 7.4; 0.15M NaCl; 1% Triton X-100) with the Roche Complete Protease inhibitor cocktail (1 tablet in 50ml conversion buffer), using new and dedicated glass potters. Substrates were divided into aliquots and either used or stored at −80°C immediately after homogenisation. Preparation and storage of substrates were carried out in a laboratory never previously used for prion research, using equipment specifically dedicated and maintaining in rigorously prion-free conditions. An homogeneous pool of substrates was prepared by mixing 5 individual substrates from 2–4 months old bank voles.

Serial automated PMCA (saPMCA) conditions and the additional procedures to minimise contamination have been described previously [23]. Briefly, 20μl of each inoculum was added to 180μl of substrate and mixed; 60μl of the reaction mixture was transferred into a 0.5ml screw cap PMCA tube (Multiply—Safecup, Sarstedt) for the first round of amplification. Tubes containing the reaction mixtures were incubated at 37°C, placed in a disk-shaped rack on the microplate horn of the sonicator (Misonix S3000) and the horn placed in a gravity oven. Water in the horn was circulated in a thermal bath at a constant temperature of 37°C. The standard sonication programme consisted of 20s sonication pulses every 30min for 48h periods. At the end of each round, the PMCA material was gently mixed and diluted 1:10 in fresh substrate ready for a further amplification step. All the inocula were amplified for 9 consecutive rounds. Manipulation of the samples and passages were carried out in a laminar flow hood, using double filter pipette tips. For the WB analysis of PMCA products, 20μl of PMCA material was digested with 100μg/ml PK (Sigma-Aldrich). Samples were shaken at 900 revolutions per minute (rpm) for one hour at 37°C. Digestion was subsequently blocked by adding 2μl of 10mM phenylmethylsulfonyl fluoride (PMSF) (Pierce Biotechnology—Thermo Fisher Scientific) to samples which were then incubated for 5min at 4°C. Ten micro-litres of NuPAGE LDS Sample Buffer (4X) and 3μl NuPAGE Sample Reducing Agent (10X) were added to each tube and samples were denatured by incubation at 90°C for 10 min Subsequently, 12μl of PK-digested PMCA samples were resolved by SDS-PAGE on 12% bis-Tris polyacrylamide gels (Invitrogen) and transferred to PVDF. Non-specific binding on the membrane was blocked with 1% powdered skim milk in PBS for one hour at room temperature and incubated with mouse anti-PrP monoclonal antibody SAF84 (Cayman Chemical). After washing, the membrane was incubated for 1h at room temperature with secondary HRP-conjugated antibody (diluted 1:30,000, ImmunoPure Peroxidase Conjugated Goat Anti-Mouse IgG (H+L) Pierce Biotechnology—Thermo Fisher Scientific) and signals were detected using SuperSignal West Femto Maximum Sensitivity Substrate (Pierce Biotechnology—Thermo Fisher Scientific).

## Results

### Histology and Immunohistochemistry

**Fig 1:**
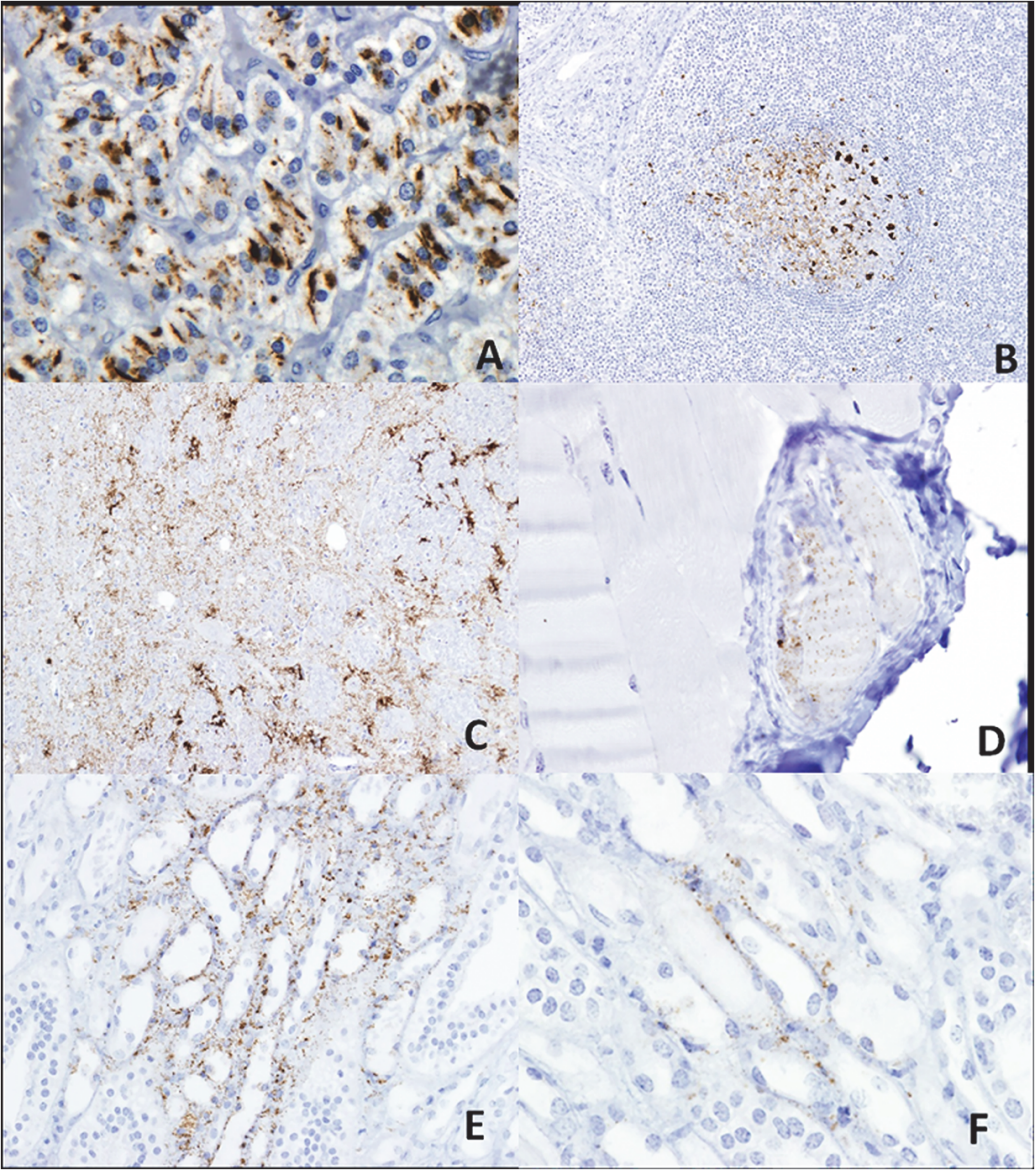
Different levels of abnormal PrP immunolabelling in selected tissues from scrapie positive sheep using R145 as primary antibody and Mayer’s haematoxylin as counterstain. A: Widespread abnormal PrP labelling in the adrenal gland from B1216 (original magnification ×20). B: Abnormal PrP in the secondary follicles of the prescapular lymph node from B1217 (original magnification ×10). C: Widespread distribution of Abnormal PrP in the obex from B1217 (original magnification ×10). D: Very localised deposition of Abnormal PrP in the spindles of the semitendinous muscle from B1216 (original magnification ×40). E and F: examples of different level of deposition of PrP^d^ in the renal papillae from respectively B1223 and B1217 (original magnification E ×20 and F×40).

All four scrapie-affected sheep examined showed severe and widespread vacuolation in the CNS and abnormal PrP was detected by IHC throughout the brain, peripheral nervous system and lymphoid tissues. Abnormal PrP was consistently detected in the adrenal glands and inconsistently detected in the kidneys and striated muscle samples (Fig. 1) but not detected in the other viscera examined.

The pattern or profile of abnormal PrP accumulation in each sheep was typical of that previously observed in ARQ/ARQ scrapie-affected Suffolk sheep in this flock [24]. The levels of vacuolation and the magnitude of the abnormal PrP profile were similar in each of the 4 sheep studied and did not assist in selection of tissues for transmission studies. Accordingly, subjectively determined levels of abnormal PrP accumulation in viscera, which were more variable than in CNS, were used to select the two sheep for transmission to bank voles.

In common with previous studies of scrapie-affected sheep in this flock [16] abnormal PrP accumulation was found in all the lymphoid tissues examined and was almost exclusively restricted to secondary follicles, more than 80% of which were positive. The only exception was the 3^rd^ eyelid, where only one sample was found to contain abnormal PrP. Lymphoid tissues of the gut showed the highest proportion of positive secondary follicles. All sheep were positive for abnormal PrP in the RAMALT which can be used for ante-mortem diagnosis of clinical and pre-clinical scrapie [25,26].

In each of the scrapie affected sheep, abnormal PrP was detected in neurons and satellite cells of all ganglia examined including those of the ENS. Abnormal PrP was also present at lower levels in the vagal and sciatic nerve trunks, where it localized within myelinated processes. In contrast to an earlier report, abnormal PrP was not detected in nerve endings of the tongue, or in sensory taste receptors [27] but, as previously described for this Suffolk flock, it was consistently present in the adrenal medulla [28]; here, it was mainly associated with the plasma-membranes of chromaffin cells, which are derived from the neural crest and are a modified post-ganglionic neuronal cell population, and to a lesser extent with peripheral nerve terminals [29]. Thus, each scrapie-affected sheep showed widespread abnormal PrP accumulation within both sympathetic and parasympathetic components of the peripheral nervous system and modified neuronal cell populations.

In each of the three striated muscles examined, abnormal PrP accumulation was confined to muscle spindles and was not detected in myocytes. Abnormal PrP was detected in two muscle spindles of the semitendinosus muscle of only one scrapie affected sheep. Muscles spindles were more frequent in the cranialis tibialis muscle (a lower hind limb muscle) and abnormal PrP positive spindles were found in two sheep. Numerous muscle spindles were present in the ocular muscle samples, and in each case abnormal PrP accumulation was detected in each muscle sample. Thus, in scrapie-affected Suffolk sheep of this flock abnormal PrP accumulation is a common feature of muscle spindles and the frequency of abnormal PrP detection in an individual muscle tissue appears to be proportionate to the number of spindles present. Abnormal PrP within muscle spindles was located in structures resembling small nerves and also within intrafusal muscle cells. In contrast, there was no evidence of abnormal PrP accumulation in motor end plates innervating myocytes.

Multiple samples of kidney were taken from each sheep and a minimum of three specifically included representation of the renal pelvis. Only two of the four sheep showed abnormal PrP accumulation in renal pelvis tissues and none showed accumulation in the cortex. This moderate frequency of abnormal PrP accumulation in the renal pelvis is consistent with previous observations in this flock and in experimental scrapie infections [30,31].

Abnormal PrP was not detected in the liver, pancreas, myocardium or lung and all tissue samples from the negative control sheep were also negative.

### Detection of abnormal PrP by biochemical tests

#### Western blot

**Fig 2:**
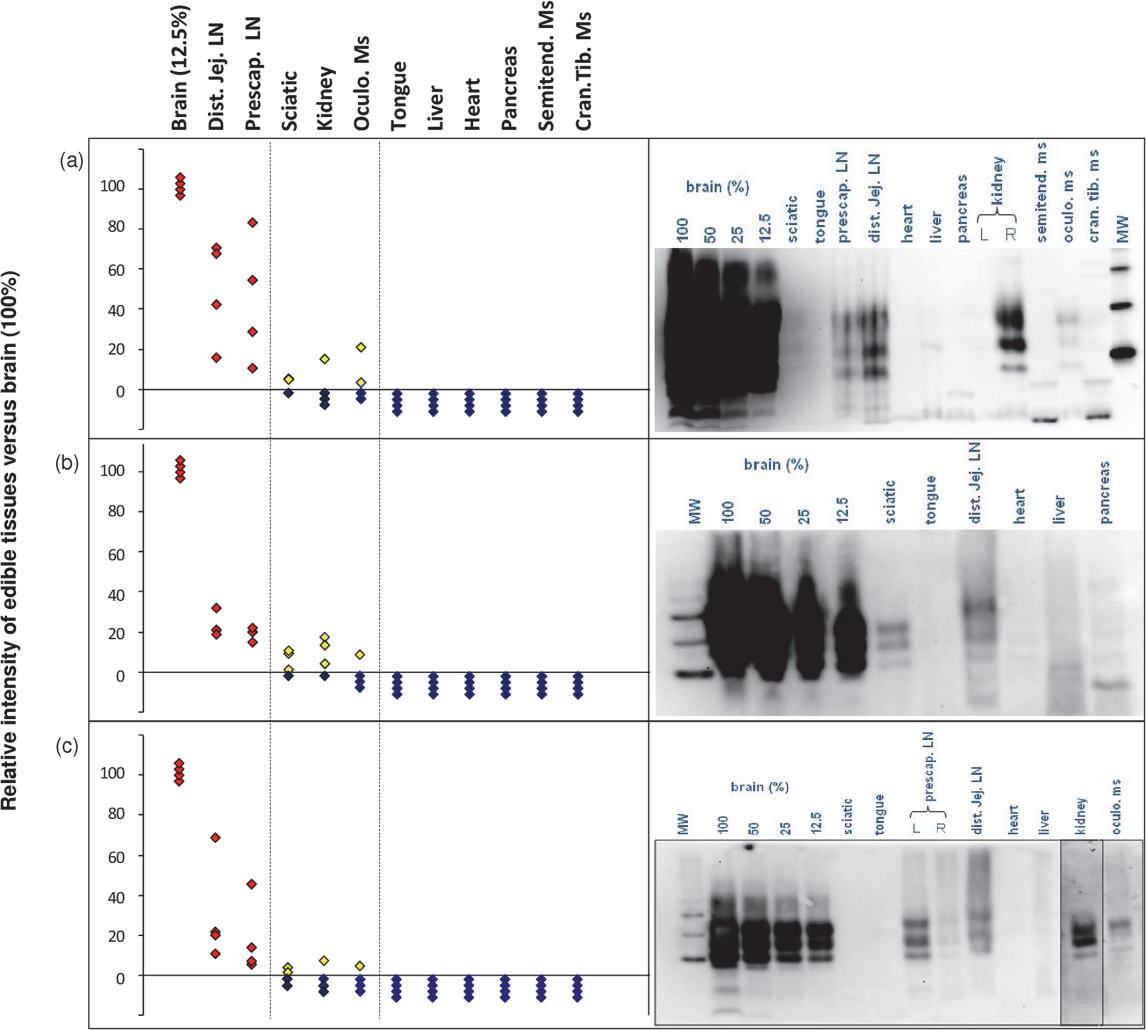
Relative intensity of chemiluminescent signal of PrPSc in tissues from 4 ARQ/ARQ sheep measured from Western blot image data (see adjacent panel) of edible tissues compared with a 12.5% dilution of brain sample by three western blot methods. (a) BioRad TeSeE Western Blot, (b) centrifugal concentration, (c) NaPTA precipitation. Red diamonds indicate high signal; yellow – low signal; blue – negative.

Abnormal PrP was consistently detected in the brain and in the prescapular and distal jejunal LNs by all three WB methods (Fig. 2). All samples of tongue, liver, heart, pancreas and semitendinosus and tibialis cranialis muscles were abnormal PrP negative by all three methods. Sciatic nerve, kidney and oculomotor muscle results varied between methods (Table 1). For example, kidney was positive for abnormal PrP in two animals by BioRad TeSeE WB but only in one using the centrifugal concentration method or NaPTA. Conversely, sciatic nerves of three sheep were positive using centrifugal concentration but only one was detected using the BioRad and NaPTA tests.

The relative WB signal intensity of each tissue was measured and expressed as a percentage of a 1/8 dilution of brain sample from the same animal (Table 1). Using the mean of the four scrapie-positive animals, all three WB methods ranked non-brain tissues in the same order, with distal jejunal LN giving the strongest signal followed by prescapular LN, sciatic nerve and kidney, with oculomotor muscle showing the lowest signal intensity.

#### ELISA

Using the BioRad TeSeE sheep and goat ELISA abnormal PrP was detected in the brain (4/4), oculomotor muscles (3/4), prescapular LN (4/4) and kidney (4/4) from the four infected sheep (Table 2). All other tissues tested were negative by this method. In some cases the signals were very low and only just above the experimentally determined cut-off point. For example the signals from the kidney and the oculomotor muscle were below 0.1 except for the left kidney from sheep 1216B. These values would fall well below the manufacturer’s cut-off value.

**Table 2:** ELISA results.

Tissue	Sheep Number									
	B1216		B1223		B1221		B1217		N523	
	BR	Idexx	BR	Idexx	BR	Idexx	BR	Idexx	BR	Idexx
**Brain**	**2.862**	**2.770**	**2.817**	**2.781**	**2.773**	**2.814**	**1.860**	**2.793**	0.006	0.018
**Prescap LN**	**1.670**	**0.822**	**1.770**	**2.816**	**1.403**	**2.598**	**2.630**	**1.892**	0.004	0.045
**Sciatic Nerve**	0.009	**0.333**	0.007	**0.538**	0.011	0.049	0.008	**0.438**	0.002	0.041
**Kidney (left)**	**0.223**	0.044	**0.048**	**1.420**	**0.045**	0.050	**0.020**	0.047	0.016	0.030
**Kidney (right)**	**0.095**	0.048	**0.028**	**0.131**	nd	0.042	**0.020**	0.059	0.015	0.063
**Oculomotor muscle**	**0.063**	**0.451**	**0.034**	0.094	0.006	**0.221**	**0.049**	**0.227**	0.005	0.031
**Tongue**	0.025	0.041	0.021	0.044	0.018	0.045	0.137*	0.032	0.013	0.035
**Heart (left ventricle)**	0.007	0.061	0.014	0.049	0.021	0.042	0.013	0.039	0.008	0.038
**Semitendinosus muscle (left)**	0.022	0.054	0.015	0.058	0.027	0.050	0.014	0.053	0.015	0.044
**Semitendinosus muscle (right)**	0.024	0.050	0.015	0.060	nd	0.044	0.015	0.050	0.011	0.045

Detection of PrP^Sc^ in target tissues from four scrapie-infected ARQ/ARQ, MRI sourced, Suffolk sheep (B1216, B1223, B1221, B1217B) and an unexposed ARQ/ARQ sheep (N523) using modified Bio-Rad TeSeE sheep and goat ELISA (BR) and the Idexx Herd-Chek scrapie antigen assay (Idexx). Values in bold are greater than the experimental cut-off values for each tissue. Tissues from the sheep underlined were inoculated into bank voles to determine infectivity. The numbers represent the absorbance values (450–620 nm). Each tissue was assayed at least twice and the results shown are values for assays completed in parallel on the same day.

Nd represents not done.

* In a repeat assay the value for this tissue fell below the cut-off value.

Using the Idexx Herd-Chek scrapie antigen assay abnormal PrP was detected in brain (4/4), oculomotor muscle (3/4), prescapular LN (4/4), kidney (1/4) and sciatic nerve (3/4) of the affected sheep (Table 2). All other tissues were negative. Largely the results of the two ELISA assays were comparable for brain, prescapular lymph node, heart, liver and semitendinosus muscle. However, the Idexx assay was able to detect abnormal PrP in sciatic nerve for which the Bio-Rad assay gave values below the cut-off value.

As described for the Western blot results we also estimated the relative levels of abnormal PrP in the positive tissues by end-point dilution using the IDEXX Herd-Chek EIA (Table 3). In all cases the brain contained the highest levels of PrP^Sc^. The prescapular lymph nodes contained on average 1.1 logs less than brain, the highest concentration of PrP^Sc^ outside the CNS. The oculomotor muscle and the sciatic nerve (where it could be measured) contained between 2.1 and 3.3 logs less respectively than the brain. Levels of PrP^Sc^ could only be measured in one kidney of one sheep with similar levels as the oculomotor muscle and sciatic nerve (in the other sheep it was undetectable by this method). This comparison in quantity can only be an estimate of relative levels of abnormal prion protein since the assays have no standard curve from which to assess the levels quantitatively.

**Table 3:** Relative quantity of PrP^Sc^ in edible tissues as measured by end-point dilution using the Idexx HerdChek antigen assay.

Tissue	Sheep Number			
	B1216	B1223	B1221	B1217
**Brain**	**1/2048**	**1/1024**	**1/2048**	**1/1024**
**Prescap LN**	**1/64**	**1/512**	**1/32**	**1/128**
**Sciatic Nerve**	**1/8**	**1/8**	nd	1/16
**Kidney (left)**	nd	**1/128**	nd	nd
**Kidney (right)**	nd	**1/2**	nd	nd
**Oculomotor muscle**	**undiluted**	nd	**1/4**	**1/4**

The values shown are the final dilution where a positive signal was observed. Nd represents not done as these tissues were negative.

#### Bioassay in bank voles

In both sheep analysed by bioassay the prescapular lymph nodes (LNs), sciatic nerves, oculomotor muscles and kidneys contained detectable levels of infectivity by bank vole bioassay (Table 4). In contrast, bank voles inoculated with heart, tongue or semitendinosus muscle from either sheep did not show clinical signs of prion disease and no abnormal PrP was found upon post-mortem processing and analyses of tissues by histopathology and Western blot (Table 4).

**Table 4:** Bioassay of edible tissues in voles.

Sheep ID	Tissue	Clinically + WB + N (surv. t. range)	Clinically—WB + N (surv. t. range)	Clinically—WB—N (surv. t. range)	Survivors* WB—N (surv. t.)	Attack rate
B1223	Sciatic Nerve	6 (218–554)	1 (487)	6 (280–600)	2 (835)	7/15
B1216	Sciatic Nerve	5 (236–697)	2 (309; 561)	6 (211–656)	7 (735)	7/20
B1223	Prescap. LN	11 (189–475)	2 (250–369)	4 (202–657)	0	13/17
B1216	Prescap. LN	8 (242–463)	2 (277; 281)	3 (298–551)	1 (805)	10/14
B1223	Kidney	1 (466)	0	10 (204–700)	6 (785)	1/17
B1216	Kidney	1 (652)	1 (670)	9 (461–767)	8 (789)	2/19
B1223	Oculomotor Ms	6 (328–405)	0	6 (340–747)	3 (833)	6/15
B1216	Oculomotor Ms	1 (204)	0	10 (228–788)	6 (830)	1/17
B1223	Semitend. Ms	0	0	12 (202–719)	4 (805)	0/16
B1216	Semitend. Ms	0	0	10 (369–699)	8 (789)	0/18
B1223	Heart	0	0	10 (375–771)	7 (805)	0/17
B1216	Heart	0	0	9 (391–645)	4 (805)	0/13
B1223	Tongue	0	0	6 (347–789)	5 (830)	0/11
B1216	Tongue	0	0	5 (211–571)	5 (831)	0/10

* Clinically healthy voles culled at the end of the experiment.

We determined the prion titre in the brains of the two sheep by end-point titration (Table 5 and S1 Fig.). The infectivity titres, calculated by the method of Spearmann and Karber [20] were 10^5.01^ i.c. ID_50_ U/g for the brain of sheep 1216B and 10^5.35^ i.c. ID_50_ U/g for the brain of sheep 1223B. Given that none of the tissues other than the brain induced 100% attack rate in voles (Table 5), their prion titre could not be derived from incubation time assay and so was estimated by direct comparison with brain titration experiments (Table 5 and S2 Fig.). By this estimation, the two sheep showed a similar magnitude of prion distribution in tissues other than brain. The prescapular LNs contained the highest levels of infectivity, approximately 2 logs less than the brain or 10^3.01^–10^3.35^ i.c. ID_50_ U/g, while scrapie prions were barely detectable in kidneys (>3 logs less than the brain). The titres in sciatic nerves and oculomotor muscles were intermediate being 2–3 logs less than the brain.

**Table 5:** Attack rates and survival times in voles inoculated with edible tissues from scrapie affected sheep in comparison to brain endpoint dilutions.

	Sheep B1223		Sheep B1216	
	Attack rate (%)	Survival time mean (SD)	Attack rate (%)	Survival time mean (SD)
Tissues				
Brain^−1^	100%	187 (13)	100%	176 (13)
Brain^−2^	100%	225 (93)	100%	212 (39)
Brain^−3^	92%	351 (93)	73%	278 (46)
Brain^−4^	23%	403 (154)	8%	216
Brain^−5^	0%		0%	
Brain^−6^	0%		0%	
Sciatic Nerve	47%	339 (153)	35%	411 (168)
Tongue	0%		0%	
Prescap. LN	76%	276 (80)	71%	317 (71)
Heart	0%		0%	
Kidney	6%	456*	11%	652–670**
Semitend. Ms	0%		0%	
Oculomotor Ms	40%	376 (28)	6%	204*

* the survival time of the only positive vole is reported

** the survival times of the two positive voles are reported

#### Detection of abnormal PrP in the inocula used for bioassay

During the project it became evident that there might have been differences between aliquots or sub-samples from the same organ or tissue. In order to compare biochemical tests and bioassay results as accurately as possible we analysed by WB all the tissue inocula prepared for the bioassay, including the different brain dilutions. For each inoculum, 3ml were analysed in 3 independent WB experiments (1ml for each WB assessment) and the results are summarised in Table 6.

**Table 6:** Comparison of PrP^Sc^, infectivity and converting activity by PMCA in the inocula.

	Sheep B1223		Sheep B1216		
	WB*	Bioassay**	WB*	Bioassay**	PMCA
Brain −1	**pos**	**pos**	**pos**	**pos**	**pos**
Brain −2	**pos**	**pos**	**pos**	**pos**	**pos**
Brain −3	**pos**	**pos**	**pos**	**pos**	**pos**
Brain −4	neg	**pos**	neg	**pos**	**pos**
Brain −5	neg	neg	neg	neg	**pos**
Brain −6	neg	neg	neg	neg	neg
Sciatic Nerve	**+**	**++**	**+**	**++**	**pos**
Tongue	−	−	−	−	neg
Prescap. LN	**++**	**+++**	**++**	**+++**	**pos**
Heart	−	−	−	−	neg
Kidney	**+/**−	**+**	−	**+**	**pos**
Semitend. Ms	−	−	−	−	neg
Oculomotor Ms	**+**	**++**	**+/**−	**+**	**pos**

* For the brain dilutions, only positive and negative dilutions are indicated. For the edible tissues a semi-quantification is provided. The WB signal was semi-quantified as follows:

+++ = a tissue positive in all experiments and with a WB signal between brain dilutions −2 and −3

++ = a tissue positive in at least 2/3 experiments and with a WB signal similar to brain dilution −3 or less

+/− = a tissue positive in 1/3 experiments and with a WB signal below brain dilution −3

** For the brain dilutions, only positive and negative dilutions are indicated. For the edible tissues a semi-quantification is provided. The bioassay results were categorised as follows (see Table 5 and S2 Fig.)

+++ = similar to brain dilution −3 or more efficient

++ = between brain dilutions −3 and −4

+ = similar to brain dilution −4 or less efficient

In both sheep chosen for bioassay, the last positive brain dilution was 10^−3^, which suggests that the WB was a log^10^ less sensitive than the bank vole bioassay (Table 6). Among the visceral tissues analysed, prescapular LNs showed the highest levels of PrP^Sc^, showing PrP^Sc^ amounts intermediate between brain dilutions 10^−2^ and 10^−3^. Clear-cut positive signals, corresponding to a concentration less than brain dilution 10^−3^ were also observed in sciatic nerves of both sheep and in the oculomotor muscles of sheep 1223B. The kidney of 1223B and the oculomotor muscle of 1216B gave unambiguous PrP^Sc^ signals in 1 out of 3 WB experiments only, while all other tissues were repeatedly negative. The comparison between the WB and bioassay results suggest a good correlation between the amount of PrP^Sc^ present and the level of infectivity as determined by bioassay (Table 6). Results of IHC, WB, ELISA and bioassay are summarised in Table 7.

**Table 7:** Summary of the IHC, WB, ELISA and bioassay results.

	Scrapie 1 (B1216)				Scrapie 2 (B1223)				Scrapie 3 (B1221)				Scrapie 4 (B1217)				Negative Control (N523)			
Tissues	IHC	WB	E	VB	IHC	WB	E	VB	IHC	WB	E	VB	IHC	WB	E	VB	IHC	WB	E	VB
**Brain**	+++	+/+/+	+/+	pos	+++	+/+/+	+/+	pos	+++	+/+/+	+/+	nd	+++	+/+/+	+/+	nd	−	− /− /−	−/−	nd
**Distal Jejunal lymph node**	+++	+/+/+	nd	nd	+++	+/+/+	nd	nd	+++	+/+/+	nd	nd	+++	+/+/+	nd	nd	−	− /− /−	nd	nd
**Prescapular lymph Node**	+++	+/nd/+	+/+	+++	+++	+/+/+	+/+	+++	+++	+/+/+	+/+	nd	+++	+/+/+	+/+	nd	−	− /− /−	−/−	nd
**Oculomotor muscle**	+++	+/+/+	+/+	+	+++	+/+/−	+/−	++	+++	− /− /−	−/+	nd	+++	+/+/+	+/+	nd	−	− /− /−	−/−	nd
**Sciatic nerve**	+	−/+/−	−/+	++	+	+/+/−	−/+	++	+	−/−/nd	−/−	nd	++	−/+/+	−/+	nd	−	− /− /−	−/−	nd
**Kidney**	−	− /− /−	+/−	+	+++	+/+/+	+/+	+	−	+/−/−	+/−	nd	++	− /− /−	+/−	nd	−	− /− /−	−/−	nd
**Semitendinosus muscle**	++	− /− /−	−/−	−	−	−/− /−	−/−	−	−	− /− /−	−/−	nd	−	− /− /−	−	nd	−	− /− /−	−/−	nd
**Cranialis tibialis muscle**	+++	− /− /−	nd	nd	−	− /− /−	nd	Nd	−	− /− /−	nd	nd	++	− /− /−	nd	nd	−	− /− /−	nd	nd
**Tongue**	−	− /− /−	−/−	−	−	− /− /−	−/−	−	−	− /− /−	−/−	nd	−	− /− /−	+/−	nd	−	− /− /−	−/−	nd
**Heart**	−	− /− /−	−/−	−	−	− /− /−	−/−	−	−	− /− /−	−/−	nd	−	− /− /−	−/−	nd	−	− /− /−	−/−	nd
**Liver**	−	− /− /−	−/−	nd	−	− /− /−	−/−	nd	−	− /− /−	−/−	nd	−	− /− /−	−/−	nd	−	− /− /−	−/−	nd
**Pancreas**	−	− /− /−	nd	nd	−	− /− /−	nd	nd	−	− /− /−	nd	nd	−	− /− /−	nd	nd	−	− /− /−	nd	nd

IHC: Score denotes the abundance of PrP^d^ in each visceral tissue; no labelling (−), mild (+), moderate(++) or severe accumulation (+++) of PrP^d^. For muscle samples a semi-quantitative scoring system was applied to no. of positively labelled spindles; 0 (−), 1 (+), 1–5 (++), >5 (+++)

WB: +1/ +2/ +3 denotes pos/neg results from three separate methods where 1 = BioRad; 2 = centrifugal concentration; 3 = NaPTA

E = ELISA: +1/ +2 denotes pos/neg results from two separate methods where 1 = BioRad; 2 = IDEXX

VB = Vole bioassay results were categorised as follows (+++) = similar to brain dilution-3 or more efficient,

(++) = between brain dilutions −3 and −4, (+) = similar to brain dilution −4 or less efficient

#### *In vitro* converting activity in the inocula used for bioassay

**Fig 3:**
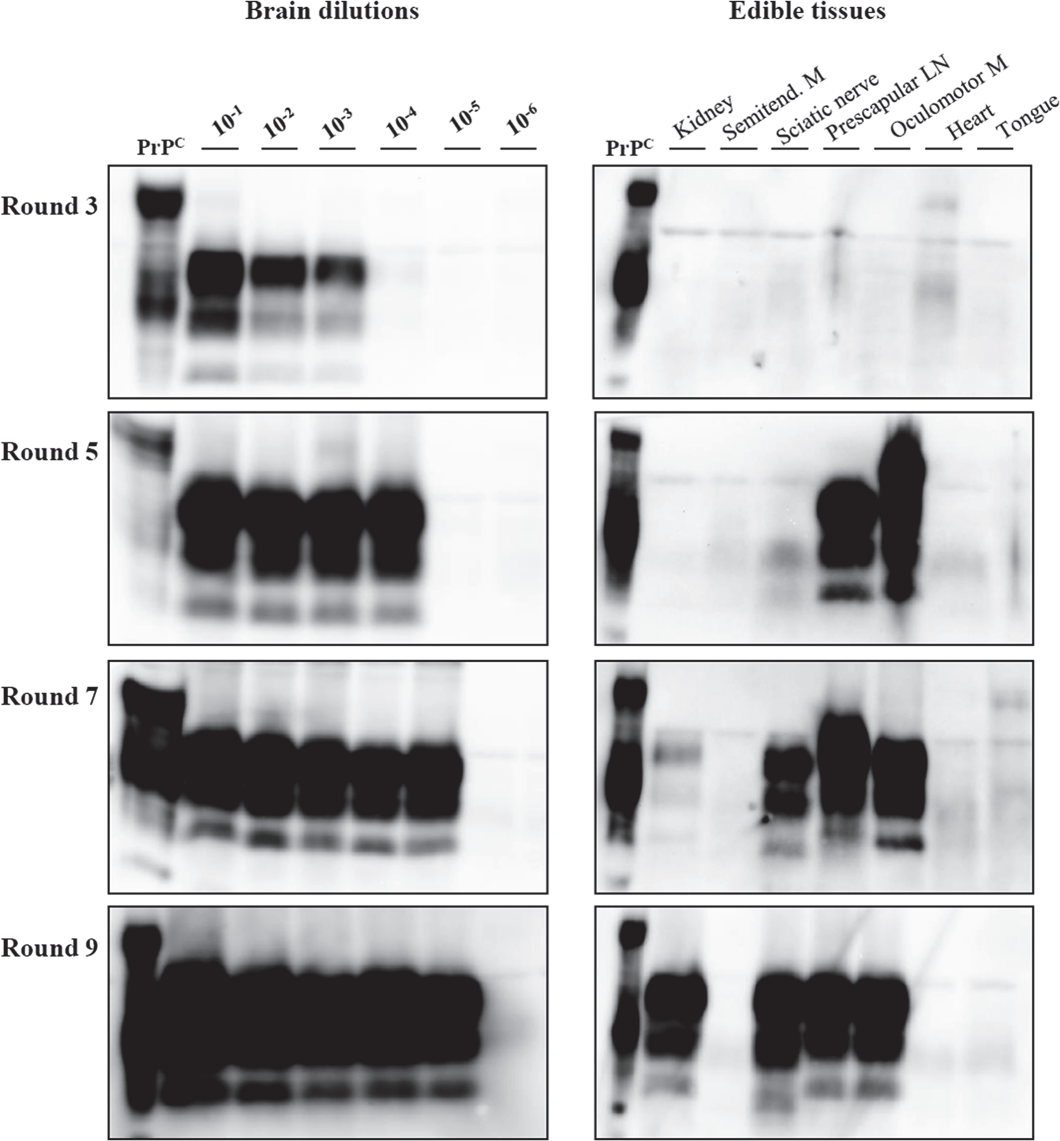
Converting activity in the inocula from 1216B sheep analysed by vPMCA. Western blot analysis of the amplified products was carried out after the 3^rd^, 5^th^, 7^th^ and 9^th^ rounds, as indicated. The results obtained with the brain dilution curve are reported in left panels, those from visceral tissue inocula are in right panels. Blots were probed with SAF84 primary antibody.

We have previously established vPMCA as a very sensitive and reliable *in vitro* conversion assay [23]. For this part of the study we used the inocula corresponding to the different brain dilutions and peripheral tissues of sheep 1216B as seeds for vPMCA and performed 9 PMCA rounds, with WB analysis after the 3^rd^, 5^th^, 7^th^ and 9^th^ rounds (Table 6 and Fig. 3). Brain dilutions up to 10^−3^ were already positive after the 3^rd^ PMCA round, reaching 10^−4^ and 10^−5^ dilutions at the 5^th^ and 7^th^ rounds, respectively; afterwards, no further converting activity was detected in higher brain dilutions up to 9 PMCA rounds. Since the 10^−^5 brain dilution was negative by bioassay, this would imply a ∼10 fold higher sensitivity of PMCA compared to bioassay (Table 6). In spite of this higher sensitivity, vPMCA only detected converting activity in the same visceral tissues which contained detectable levels of infectivity by bank vole bioassay, i.e. sciatic nerve, prescapular lymph node, kidney and oculomotor muscles (Table 6 and Fig. 3). In these tissues, vPMCA-converted abnormal PrP was detected after 5 (oculomotor muscles and prescapular lymphnode) or 7 (sciatic nerve and kidneys) vPMCA rounds, while tissues that were negative by bioassay (heart, tongue and semitendinosus muscle) remained negative up to the 9^th^ vPMCA round (Table 6 and Fig. 3). Eight unseeded vPMCA reactions run in parallel as negative controls remained negative up to the 9^th^ vPMCA round (S3 Fig.).

## Discussion

We have studied the distribution of scrapie infectivity and Abnormal PrP in 4 ARQ/ARQ sheep naturally affected with scrapie with a neuropathological phenotype indistinguishable from other scrapie cases in sheep of the same genotype from different European countries [13]; these previous studies have reported the tissue distribution of PrP^Sc^ by IHC but have limited biochemical and bioassay data [16,14,32]. We have found detectable levels of infectivity and/or abnormal PrP in all 4 sheep in some of the edible tissues, such as skeletal muscles and kidneys, or anatomical structures dispersed within them, such as lymph nodes, nerves and autonomic ganglia. However, no evidence of infection was found in the heart, tongue, pancreas and liver from any of the sheep. The detection of infectivity and abnormal PrP in kidneys corroborates previous IHC-based reports [31,33], while our negative findings in the tongue are in contrast with a study which detected abnormal PrP in this tissue [27]. Although the infectivity load in the kidney was low, >3 logs less than the brain, this finding is important as kidneys are not currently categorised as SRM and thus enter the human and pet food chain. Aside from this, the findings in the present and previous studies [31,33,34] demonstrating that scrapie is present in tissues which produce excretions/secretions such as kidneys and salivary glands, complement studies in wild ruminants with CWD [35] and emphasize the potential role of urine and saliva in the environmental spread of TSEs.

Regarding skeletal muscles, which are quantitatively the most important edible tissue in terms of human exposure to prions, our findings corroborate previous data from VRQ/VRQ sheep [36,37]. In agreement with Andreoletti and co-workers (2004), we found IHC-detectable abnormal PrP exclusively in muscle spindles, which are innervated structures involved in proprioception. Their number varies markedly among individual muscles, being least frequent in the large muscles of the hind limb and much more frequent in muscles involved in fine motor control, such as the intra-orbital muscles. Significantly, by analysing different type of muscles we found that the frequency of muscle spindles roughly correlated with the probability of detecting abnormal PrP and with the infectivity load, which has obvious implications for risk modelling of infectivity entering the human food chain. However, even muscles with high spindle content and that were positive by laboratory tests, such as the oculomotor muscles, harboured relatively low levels of infectivity per gram of tissue, i.e. ∼3 logs less than the brain and ∼1–2 logs less than the lymph nodes. These results are in contrast with those obtained from a single VRQ/VRQ sheep with natural scrapie [37], where skeletal muscles had infectious loads similar to lymphoid tissues and only ∼1 log less than the brain. It is not certain if these discrepancies are due to the sheep genotype, the scrapie strain involved or to technical aspects such as tissue sampling, which indeed could result in significant bias given the uneven distribution of abnormal PrP observed in skeletal muscles.

Our negative findings in the liver are discrepant also from the results of a study with VRQ/VRQ sheep [38]. In this latter study, 89% of sheep with scrapie were positive for abnormal PrP in the liver by both ELISA and WB techniques similar to those used in the present study, suggesting that technical aspects are not an explanation for the differences observed between ARQ/ARQ and VRQ/VRQ sheep. Overall, these observations suggest that the prion strain and the sheep genotype independently or together have a significant effect on the risk associated with liver and muscle and indeed, in general, on the tissue distribution of the scrapie agent.

Beyond the general agreement between the different tests, some discrepancies in sensitivity were found between the three WB methods, as well as between the two ELISAs. These where evident where low titres of infectivity were present, for example in sciatic nerves, where centrifugal concentration followed by WB and the Idexx antigen assay were more sensitive than the other laboratory methodologies, or in kidneys, where BioRad TeSeE WB and ELISA were the most sensitive tests. One possible explanation, supported by the similar observations comparing the Bio-Rad TeSeE WB and ELISA that share the same tissue preparation method, could be that different sample preparations might selectively favour the release of PrP^Sc^ from different tissues. On the other hand, IHC results showed that PrP^Sc^ was present in specific locations within the kidneys and muscles and a high proportion of the tissue was negative, therefore gross sampling post-mortem may not have incorporated an area with PrP^Sc^ present. Furthermore, the amount of PrP^Sc^ within these tissues appears to be close to the detection limit of laboratory tests and the final result may be reliant on the size of the starting sample. Overall, these observations suggest that the focal and sparse accumulation of abnormal prion protein observed in this study could have important implications for risk modellers, as it exemplifies how difficult it could be to extrapolate mean data, such as ID50 per gram of tissue, from experimental data generated from a low number of tissue homogenates derived from relatively little starting material.

Importantly, in this study we looked for abnormal PrP and infectivity in the same tissue samples that had been analysed by IHC. This approach offered a good perspective to compare the results obtained, to derive meaningful correlations between abnormal PrP and scrapie infectivity and to explain potential pitfalls of tissue homogenate-based approaches to infectivity estimations. Overall, we observed a good correlation between the presence and relative quantity of abnormal PrP and the presence and titre of infectivity in a given tissue. Indeed, by combining the results obtained with the different ELISA and WB techniques, we found that their predictive value was high, as only those tissues which were positive by one or more laboratory method were positive by vole bioassay. This was further confirmed by WB detection of PrP^Sc^ directly in the tissue homogenates used for bioassay in voles. Even more interesting, quantification of PrP^Sc^ by WB or ELISA showed the same rank order of positivity as bioassay, with lymph nodes (1-to-2 logs less than the brain) > sciatic nerves (2-to-3 logs less than the brain) ≥ oculomotor muscles and kidneys.

At first glance these conclusions would appear to contradict a previous study in which the authors determined correlations among biochemical tests, bioassay and IHC [39]. However, that study was concerned with brain regions and the distinct morphological types of abnormal PrP observed therein. Significant heterogeneity is observed in the patterns of abnormal PrP staining in the brain by IHC with regard to association with cell type, morphology and whether the PrP is present as extracellular or intracellular particles. The regions evaluated in that study were chosen to assess how well they correlate with the biochemical tests and the bioassay. In the current study the brain samples are not distinguished based on these categories and with regard to subtypes are likely to be more homogeneous than those in the previous study. Furthermore the cell types associated with abnormal PrP and the type of staining observed in visceral tissues, within an individual tissue, are much less heterogeneous than that found among different brain regions. Thus the findings from the two studies are not necessarily discordant. In addition, the use of a different model for bioassay (a transgenic mouse for ovine ARQ PrP) and focus on BSE as the infectious agent may also account for differences in results.

In conclusion, our findings confirm and extend previous knowledge on the presence of infectivity within the edible tissues of sheep with scrapie, and suggest that laboratory tests may be exploited to extrapolate the infectivity titre in tissues derived from ARQ/ARQ sheep. Given the sensitivity observed with PMCA, which was even more sensitive than bioassay, and the recent advances made by several groups in scaling down protocols for quantitative PMCA [40,41], this technique might be seen as a promising candidate to be optimised for a highly sensitive quantitative assay.

## Supporting Information

S1 Fig Titration of infectivity in sheep brains by end point dilution in bank voles. Serial dilutions of brain homogenates from sheep 1216B (right panel) and sheep 1223B (left panel) scrapie isolates were inoculated intracerebrally into bank voles. Symbols represent individual survival times. The diseased voles were positive for brain PrP^Sc^. Inoculated voles that were negative after 868 d.p.i. (right panel) or 862 d.p.i (left panel) are plotted in compressed form after the *x* axes break point. Voles culled with intercurrent disease at >200 d.p.i. and negative by WB are plotted as survivors. The mean survival time (days ± sd) and the number of diseased/inoculated voles are indicated on the right of each chart. Both end-point titrations gave similar results, in that the 10^−1^ and 10^−2^ dilutions gave 100% attack rate, the 10^−3^ and 10^−4^ dilutions gave attack rates lower than 100%, while higher dilutions were unable to infect any inoculated vole. The infectivity titres, calculated by the method of Spearmann and Karber, were 10^5.01^ i.c. ID_50_ U/g for the brain of sheep 1216B and 10^5.35^ i.c. ID_50_ U/g for the brain of sheep 1223B. These results are in line with those previously published for brain tissues from sheep affected by the same scrapie strain used in this project [13].
(TIF)

S2 Fig Survival curves obtained from positive edible tissues of sheep 1216B and 1223B in comparison with the respective brain dilutions. For sheep 1216B (top panel) the prescapular LN gave a survival curve very similar to that of brain dilution 10^−3^, the sciatic nerve was intermediate between dilutions 10^−3^ and 10^−4^, while oculomotor muscle and kidney were similar to a brain dilution of 10^−4^ or less. For sheep 1223B (bottom panel) the prescapular LN gave a survival curve very similar to that of brain dilution 10^−3^, the sciatic nerve and oculomotor muscle were intermediate between dilutions 10^−3^ and 10^−4^, while the kidney showed a survival rate higher than brain dilution 10^−4^.
(TIF)

S3 Fig Converting activity in unseeded negative controls analysed by vPMCA in parallel with reactions seeded with sheep brain dilutions and edible tissues (shown in Fig. 3). The western blot analysis of the amplified products carried out after the 9^th^ vPMCA round shows absence of converting activity in negative controls. Blots were probed with SAF84 primary antibody.
(TIF)
