# Supplementary figures and images for: Correlation between Infectivity and Disease Associated Prion Protein in the Nervous System and Selected Edible Tissues of Naturally Affected Scrapie Sheep
Source: PLoS One. 2015 Mar 25;10(3):e0122785. doi: 10.1371/journal.pone.0122785 (PMC4373927; doi:10.1371/journal.pone.0122785)

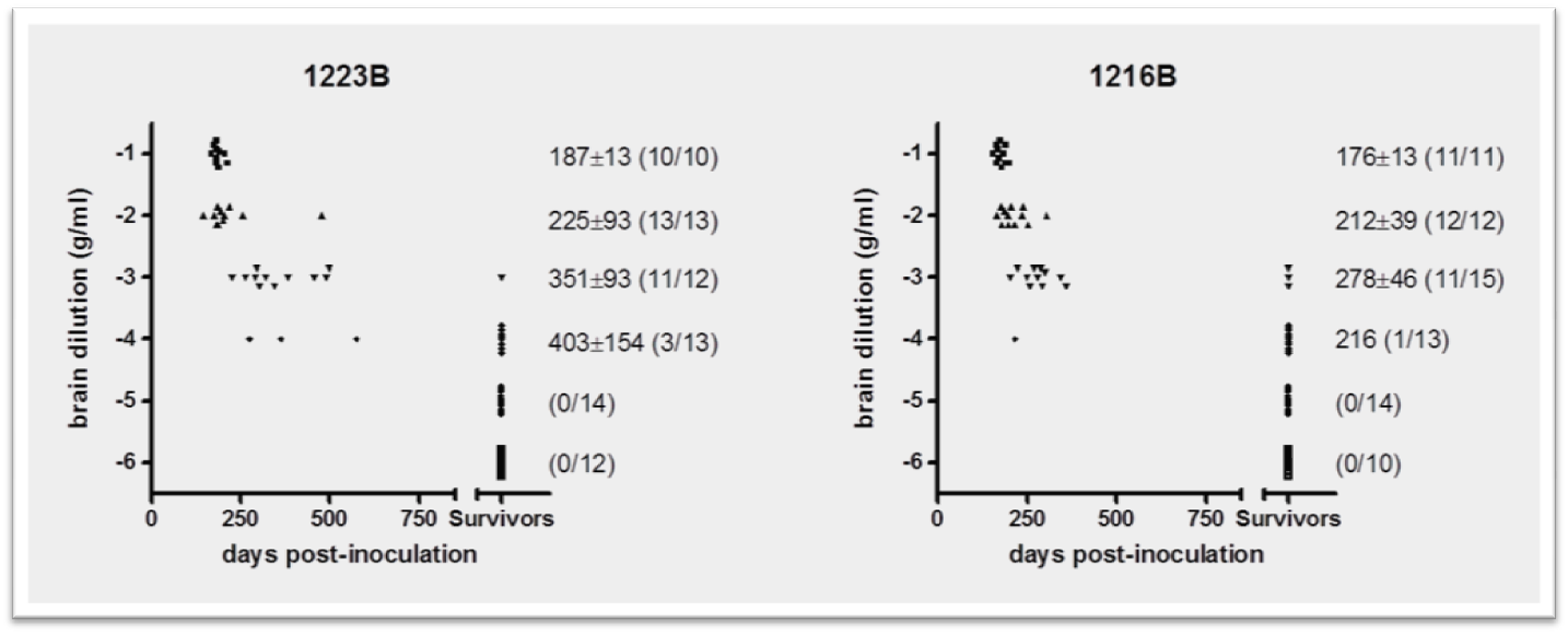

Supplement: S1 Fig — Serial dilutions of brain homogenates from sheep 1216B (right panel) and sheep 1223B (left panel) scrapie isolates were inoculated intracerebrally into bank voles. Symbols represent individual survival times. The diseased voles were positive for brain PrPSc. Inoculated voles that were negative after 868 d.p.i. (right panel) or 862 d.p.i (left panel) are plotted in compressed form after the x axes break point. Voles culled with intercurrent disease at >200 d.p.i. and negative by WB are plotted as survivors. The mean survival time (days ± sd) and the number of diseased/inoculated voles are indicated on the right of each chart. Both end-point titrations gave similar results, in that the 10−1 and 10−2 dilutions gave 100% attack rate, the 10−3 and 10−4 dilutions gave attack rates lower than 100%, while higher dilutions were unable to infect any inoculated vole. The infectivity titres, calculated by the method of Spearmann and Karber, were 105.01 i.c. ID50 U/g for the brain of sheep 1216B and 105.35 i.c. ID50 U/g for the brain of sheep 1223B. These results are in line with those previously published for brain tissues from sheep affected by the same scrapie strain used in this project [13]. (TIF) [file pone.0122785.s001.tif]

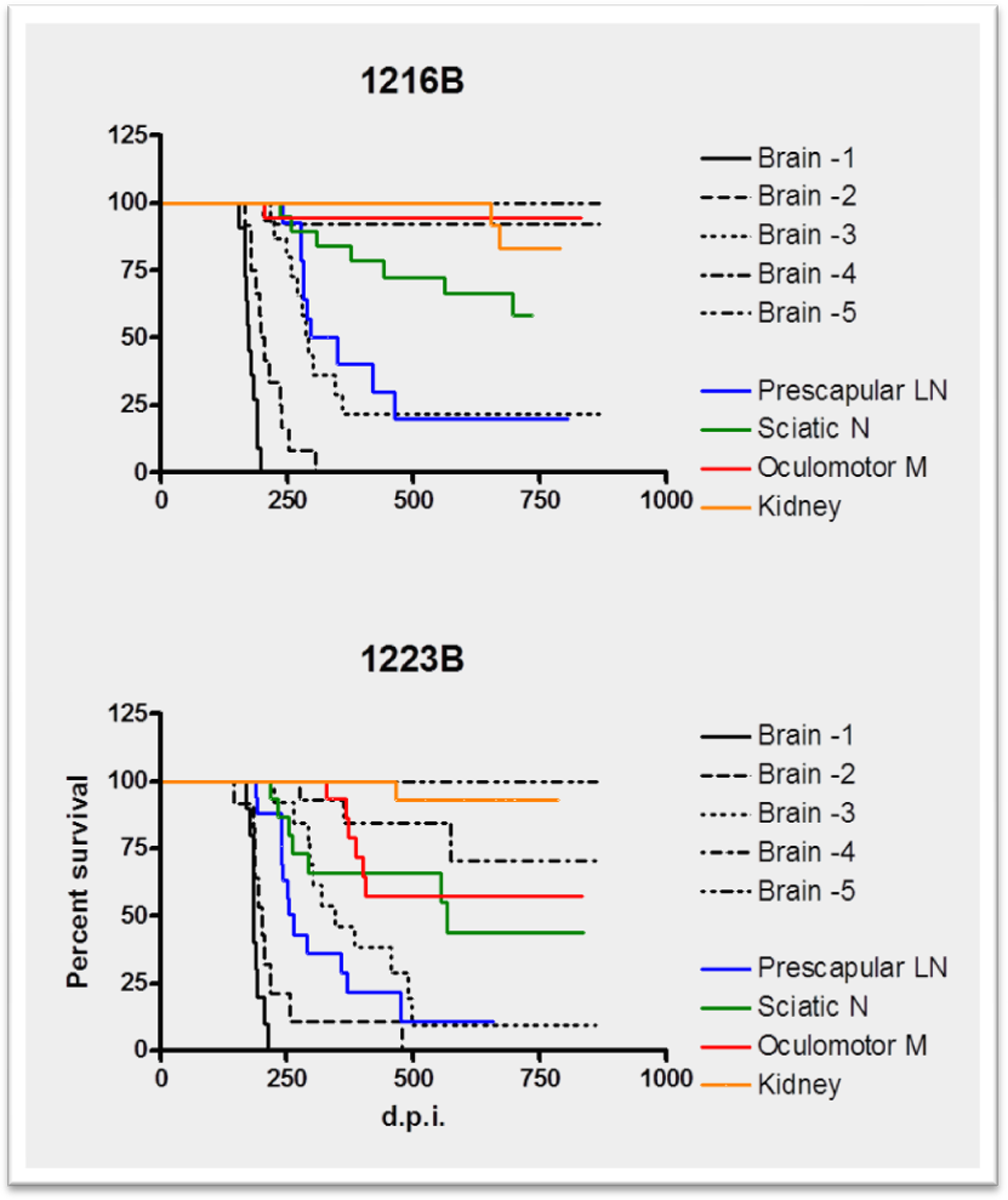

Supplement: S2 Fig — For sheep 1216B (top panel) the prescapular LN gave a survival curve very similar to that of brain dilution 10−3, the sciatic nerve was intermediate between dilutions 10−3 and 10−4, while oculomotor muscle and kidney were similar to a brain dilution of 10−4 or less. For sheep 1223B (bottom panel) the prescapular LN gave a survival curve very similar to that of brain dilution 10−3, the sciatic nerve and oculomotor muscle were intermediate between dilutions 10−3 and 10−4, while the kidney showed a survival rate higher than brain dilution 10−4. (TIF) [file pone.0122785.s002.tif]

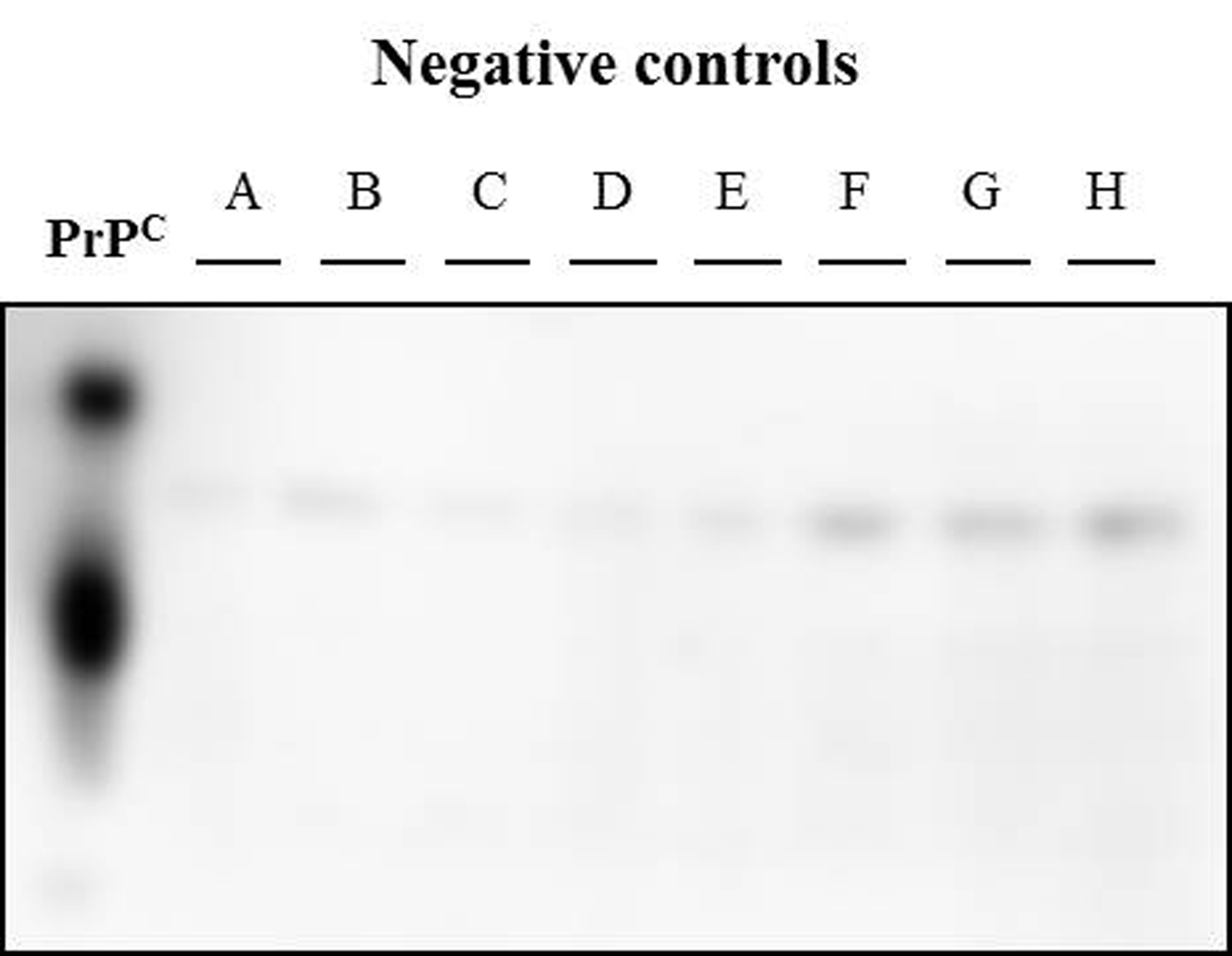

Supplement: S3 Fig — The western blot analysis of the amplified products carried out after the 9th vPMCA round shows absence of converting activity in negative controls. Blots were probed with SAF84 primary antibody. (TIF) [file pone.0122785.s003.tif]
